# The Use of Boiled Drinking Water, and Calcification in Childhood and Youth

**Published:** 1911-03-15

**Authors:** J. Ferrier


### THE USE OF BOILED DRINKING WATER, AND CALCIFICATION IN CHILDHOOD AND YOUTH.

BY DR. J. FERRIER.

Dental caries is observed at its maximum frequency in adolescence. The histogenesis of the teeth greatly varies in different individuals, so that it may be said that each tooth carries in itself its chances for longevity or early doom. The greatest danger for the tooth exists at the period when the severest stress is brought to bear upon the whole system of the youth in building up the osseous frame, viz, at the age from six to fifteen years, when the youthful individual is generally hypocalcified, and the dental tissues are most likely to be interfered with in their formative development by bacterial invasion. Normally, in healthy adults, caries progresses less rapidly or become rare, a certain stage of comparative immunity being reached. In looking for the cause of an onset of rapidly progressing caries in otherwise healthy adults and in well-cared for and normal youths, Ferrier found a striking coincidence of caries with the habitual use of boiled drinking water, which has been deprived of all its calcium carbonate. These findings are indorsed by Professor Armand Gautier's statement that youth during the period of the formation of the osseous frame requires

0.825 gram of calcium daily. Since the average diet furnishes but 0.765 gram, the missing 0.65 gram must be supplied by calciferous drinking water. To prove this theory, Gautier experimented on three young pigs, the deficient osseous development of which, as observed after their being killed, corresponded exactly to the lack of calcium salts in the animal's drinking water. It is evident there from that during the period of stress, *i. e.* adolescence, when there is already a tendency to hypocalcification, the lack of calciferous drinking water will affect the teeth as well as the other osseous structures. In the osseous frame this faulty diet may produce nothing more serious than slender and light build, while the teeth will lack in resistance to the ravages of caries and its serious consequences.

In pointing out the close relationship between hypocalcification and susceptibility to tuberculosis, Ferrier cites a number of feeding experiments in rabbits and guinea-pigs as reported in medical literature, which showed a correspondingly greater resistance to tubercular infection as they were fed on foods rich or poor in calcium salts. Paul Ferrier and others have also shown that an increase in calciferous foods, liquid and solid, increases the chances of recovery of tubercular patients.

In summing up, the author makes a plea for the habitual use of unboiled drinking water, and holds it up as the duty of public authorities, parents, public institutions and schools to provide for pure drinking water.—*Cosmos.*

